# Toll-Like Receptor 4 Reduces Oxidative Injury via Glutathione Activity in Sheep

**DOI:** 10.1155/2016/9151290

**Published:** 2015-11-10

**Authors:** Shoulong Deng, Kun Yu, Qian Wu, Yan Li, Xiaosheng Zhang, Baolu Zhang, Guoshi Liu, Yixun Liu, Zhengxing Lian

**Affiliations:** ^1^State Key Laboratory of Reproductive Biology, Institute of Zoology, Chinese Academy of Sciences, Beijing 100101, China; ^2^Laboratory of Animal Genetics and Breeding, College of Animal Science and Technology, China Agricultural University, Beijing 100193, China; ^3^State Key Laboratory of Agrobiotechnology, College of Biological Sciences, China Agricultural University, Beijing 100193, China; ^4^School of Biological Science and Medical Engineering, Beijing University of Aeronautics and Astronautics, Beijing 100191, China; ^5^Tianjin Institute of Animal Sciences, Tianjin 300112, China; ^6^State Oceanic Administration, Beijing 100860, China

## Abstract

Toll-like receptor 4 (TLR4) is an important sensor of Gram-negative bacteria and can trigger activation of the innate immune system. Increased activation of TLR4 can lead to the induction of oxidative stress. Herein, the pathway whereby TLR4 affects antioxidant activity was studied. In TLR4-overexpressing sheep, TLR4 expression was found to be related to the integration copy number when monocytes were challenged with lipopolysaccharide (LPS). Consequently, production of malondialdehyde (MDA) was increased, which could increase the activation of prooxidative stress enzymes. Meanwhile, activation of an antioxidative enzyme, glutathione peroxidase (GSH-Px), was increased. Real-time PCR showed that expression of activating protein-1 (AP-1) and the antioxidative-related genes was increased. By contrast, the expression levels of superoxide dismutase 1 (SOD1) and catalase (CAT) were reduced. In transgenic sheep, glutathione (GSH) levels were dramatically reduced. Furthermore, transgenic sheep were intradermally injected with LPS in each ear. The amounts of inflammatory infiltrates were correlated with the number of TLR4 copies that were integrated in the genome. Additionally, the translation of *γ*-glutamylcysteine synthetase (*γ*-GCS) was increased. Our findings indicated that overexpression of TLR4 in sheep could ameliorate oxidative injury through GSH secretion that was induced by LPS stimulation. Furthermore, TLR4 promoted *γ*-GCS translation through the AP-1 pathway, which was essential for GSH synthesis.

## 1. Introduction

Toll-like receptor 4 (TLR4) is a pattern-recognition receptor (PRR) that plays a key role in innate immunity and host defense. TLR4 is a critical signal transducer of lipopolysaccharide (LPS), the major exocellular component of Gram-negative bacteria. The activation of TLR4 can promote cell proliferation and apoptosis [1]. TLR4 can initiate immune responses through both myeloid differentiation primary response gene 88- (MyD88-) dependent and independent pathways. In the MyD88-dependent pathway, TLR4 activates nuclear factor-*κ*B (NF-*κ*B) and activating protein-1 (AP-1), which leads to oxidative stress and inflammation [2, 3].

Oxidative stress was frequently observed after pathogenic microbial infections. In this condition oxidative stress is supposed to ward off pathogenic microbes. When excessive, tissue can be damaged by the overproduction of reactive oxygen species (ROS) and reactive nitrogen species (RNS). To protect organs, ROS/RNS are scavenged via antioxidant mechanisms. There are two major physiological antioxidant defense systems, endogenous antioxidants (glutathione) and membrane-protecting enzymes (superoxide dismutase (SOD), catalase (CAT), and glutathione peroxidase (GSH-Px)) [4]. Genes, such as CAT, SOD, glutathione S transferase (GST), *γ*-glutamylcysteine synthetase (*γ*-GCS), and heme oxygenase l (HOl), are used for monitoring antioxidant procedure, for their encode products related to antioxidative stress responses that protect cells from oxidative stress [5–7].

TLR4 pathways are crosslink to oxidative stress. After TLR4 triggers NF-*κ*B activation, inflammatory factors, such as interleukin-6 (IL-6) and tumor necrosis factor-alpha (TNF-*α*), are secreted. These inflammatory factors accelerate the inflammatory response by reducing SOD activity and increasing malondialdehyde (MDA) production. As a result, the increasing of ROS/RNS production could affect the antioxidative capacity of cells [8, 9]. The AP-1 pathway is involved in regulation of GSH production [10]. As an antioxidant, GSH plays an important role in preventing oxidative damage by directly interacting with ROS/RNS or by operating as a cofactor for various enzymes [11]. Being a rate-limiting enzyme, *γ*-GCS has impact on GSH synthesis. The upregulation of *γ*-GCS can increase antioxidant capacity [12].

Many sheep diseases are closely related to increased amounts of oxidation products, because accumulation of oxidative products can reduce sheep immunity and host defense responses [13]. Studies have shown that the inflammatory response is suppressed in TLR4-mutant mice [14, 15]. Overexpression of TLR4 amplifies the host response to LPS and provides transgenic mice with a survival advantage [16]. The enhanced inflammatory response helps to remove pathogens, but excessive inflammation can result in oxidative damage. Herein, we generated lines of transgenic sheep that overexpressed TLR4 with a variety of copy numbers. LPS was administered to induce oxidative damage. The TLR4 pathway, which is involved in antioxidative damage, was studied to elucidate the antioxidative stress response in sheep.

## 2. Materials and Methods

### 2.1. Genotyping of Tg Sheep

Transgenic sheep were produced by microinjection. The transformed exogenous genes in the experimental offspring were analyzed by Southern blotting of genomic DNA from ear biopsies, and we used a PCR-based method to generate a specific Digoxigenin-labeled probe (Roche Diagnostics, Mannheim, Germany). Exogenous TLR4 was analyzed by Southern blotting with the probe cTLR4 (Table 1). Genomic DNA (20 ng/*μ*L) was digested with* Vsp*I and* Sma*I (NEB, Beverly, MA, USA). The gene expression levels and TLR4 copy numbers were quantified by real-time PCR. Mononuclear cells were isolated from transgenic sheep peripheral blood using sheep lymphocyte separation medium (TBD, Tianjin, China). Real-time PCR was used to detect exogenous copies of TLR4. Primers were designed to target sequences located in cTLR4. *β-actin* was used as an internal standard (Table 1). Real-time PCR reactions were carried out with a Real Master Mix SYBR Green Kit (Tiangen, China) using MX300P (Stratagene) following the manufacturer's protocol.

### 2.2. Monocyte Cultures

Sheep with exogenous TLR4 copies ranging from 1 to 3 copies were randomly selected. Those sheep were divided into three groups based on the number of exogenous TLR4 copies. Each group included three transgenic sheep with the same number of exogenous TLR4 copies. A total of 10 mL peripheral blood from 6-month-old sheep was collected, and heparin was used for anticoagulation. Monocyte isolation and culture was carried out according to the sheep lymphocyte separation medium manufacturer's instructions. Then, 1 × 10^5^ cells were seeded in each well of 6-well plates in triplicate wells for each group. RM1640 (Gibco, Grand Island, NY, USA) medium containing 10% FBS (Gibco) was changed every 24 h. Monocytes were stimulated using LPS (1 *μ*g/mL; Sigma-Aldrich, St. Louis, MO, USA) after 48 h.

At 8 h after stimulation, culture medium was collected and frozen. RNA from adherent cells was isolated using TRIzol (Invitrogen, Carlsbad, CA, USA). Then, cDNA were synthesized. The mRNA transcript abundance of TLR4 was measured using real-time PCR. Gene expression levels of AP-1, SOD1, CAT, GST*α*1, *γ*-GCS, and HO1 were quantified by RT-PCR. Primers sequences are shown in Table 1.

### 2.3. Measurements of Oxidative Stress-Related Enzymes

Cell suspensions were dropped in liquid nitrogen and samples were thawed on ice; this freeze/thaw cycle was repeated two more times. The activities of iNOS, T-SOD, CAT, COX-2, NADPH oxidase, MDA, GSH, and GSSH were examined by spectrophotometry using respective detection kits (Jiancheng, Nanjing, China). GSH-Px was detected using an enzyme-linked immunosorbent assay (ELISA) kit (CUSABIO, Hubei, China) following previously published methods. The OD ratio was obtained using a microplate spectrophotometer.

### 2.4. *In Vivo* Injection of LPS

Local inflammatory responses were observed in three 3-month-old transgene-positive individuals after intradermal injection of LPS (3 mg/mL in 100 *μ*L) in each ear. At 8 h after challenge, tissues were collected. Pathological changes were observed using hematoxylin and eosin staining. Immunohistochemically stained sections were used for observations of *γ*-GCS (Abcam, Cambridge, UK) protein expression.

### 2.5. Statistical Analyses

All the above experiments were repeated 3 times. Data were subjected to analysis of variance using the GLM procedures of Statistical Analysis System (SAS Institute, Cary, NC, USA). All data were expressed as mean ± SEM. Differences were considered to be significant when *p* < 0.05.

## 3. Result

### 3.1. Overexpression of TLR4 Increases Oxidative Damage in Sheep

**Figure 1:**
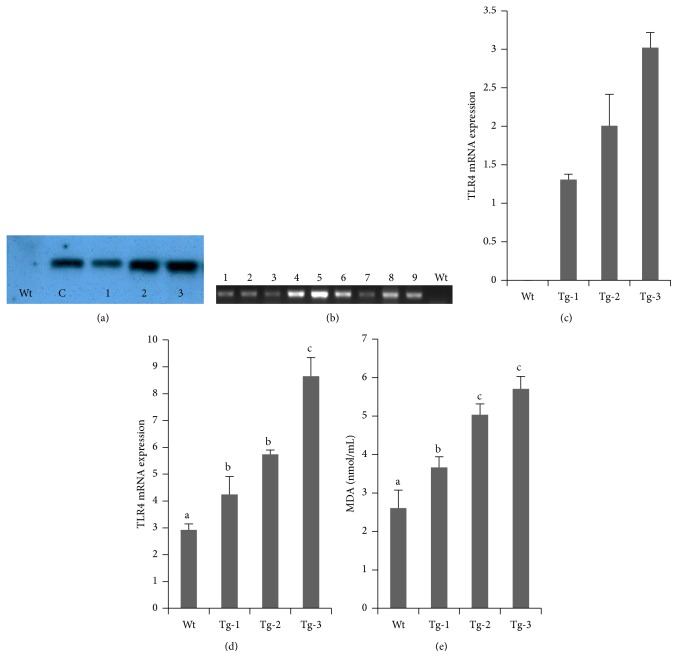
Oxidative damage in sheep with transgenic TLR4 overexpression. (a) Southern blot analysis; Wt, wild type; 1–3 were transgenic sheep (Tg); (c) TLR4 plasmid. (b) Real-time PCR product analysis by agarose gel electrophoresis. TLR4 expression levels were different in transgenic animals; 1–9, transgenic sheep (Tg). (c) Exogenous TLR4 copies were measured by real-time PCR. (d) Relative quantitative analysis of TLR4 expression in cells from transgenic sheep after LPS stimulation. (e) MDA constant measurements. Data represent mean ± SE. ^a,b,c^Different superscript letters indicate significantly different values between groups (*p* < 0.05). Wt, wild type; Tg-1, Tg-2, and Tg-3 were transgenic sheep that had 1, 2, or 3 copies of TLR4.

Transgenic sheep that overexpressed TLR4 were produced by microinjection. Southern blot analyses were used to detect positive transgenic lambs (Figure 1(a)). By analyzing the developed blots to calculate the transgene copy number, individuals were identified that carried various copy numbers of the exogenous TLR4 gene. Real-time PCR was used to study exogenous TLR4 expression. The mRNA transcript levels of monocytes from selected lambs were observed (Figure 1(b)), and the numbers of exogenous TLR4 copies were calculated, which were 1.24, 1.30, 1.38, 1.67, 1.89, 2.46, 2.84, 3.00, and 3.23, respectively. Based on the number of exogenous TLR4 copies, nine lambs were selected and divided into three groups (Figure 1(c)).

At 8 h after LPS stimulation, TLR4 transcript levels in monocytes from nine lambs were measured. We found that the TLR4 mRNA transcript levels and MDA contents were correlated with the number of exogenous TLR4 copies (Figures 1(d) and 1(e)). Transcript levels in transgenic sheep were all significantly higher than those in wild-type (Wt) sheep (*p* < 0.05). Compared with Wt sheep, more oxygen free radicals were generated, resulting in enhanced oxidative damage in transgenic animals.

**Figure 2:**
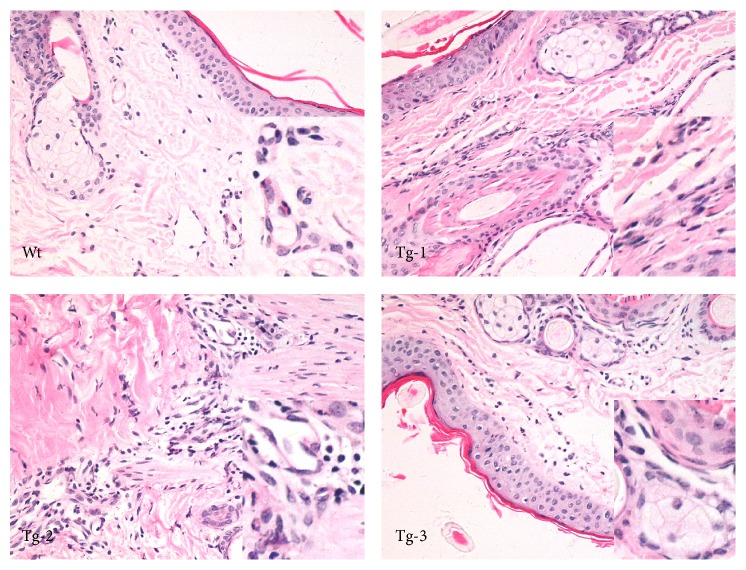
Inflammatory reactions in TLR4 transgenic sheep. Pathological changes were observed after the administration of LPS (hematoxylin and eosin staining; 400x magnification). Wt, wild type; Tg-1, Tg-2, and Tg-3 were transgenic sheep that had 1, 2, or 3 copies of TLR4.

Ear tissue inflammatory reactions induced by LPS stimulation were next assessed in Wt and transgenic sheep (Figure 2). Inflammatory cell infiltrates were observed in the dermis. Inflammatory cells were found to increase in Tg-1 group animals. Many inflammatory cells were also observed in Tg-2 animals. In Tg-3 animals, the horny layer (of the epidermis) cuticle off and many inflammatory cells, including segmented cells, could be observed. Oxidative stress induced tissue disequilibrium. Subsequently, inflammatory cell infiltration and inflammatory mediator release were detected in inflammatory tissues.* In vivo* observations of tissue sections showed that the degree of inflammation was associated with the transgene copy number.

### 3.2. TLR4 Promotes Oxidative Stress-Related Enzyme Activation following LPS Stimulation in Sheep

**Figure 3:**
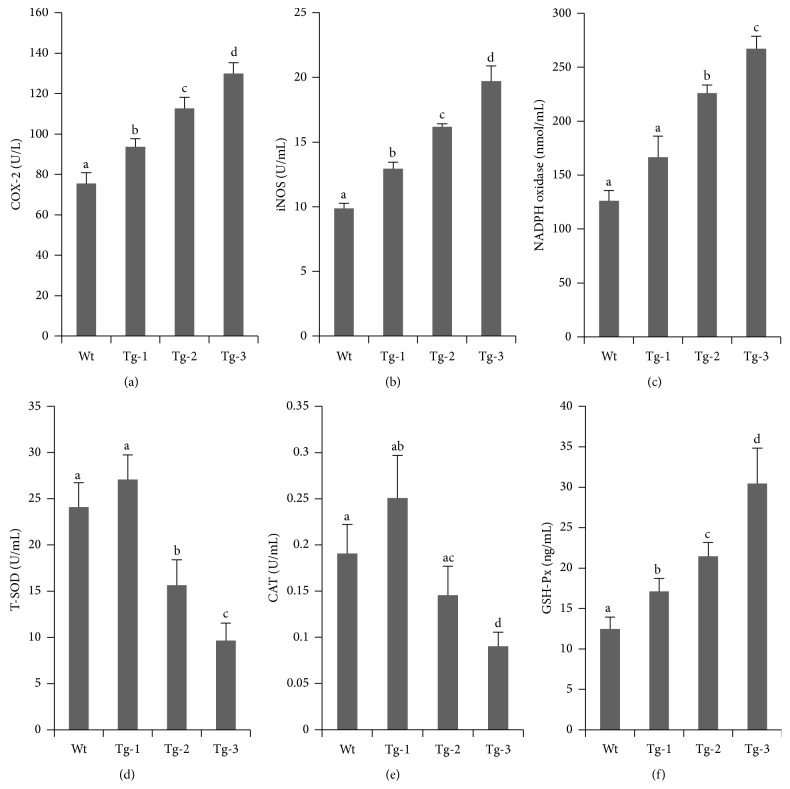
Expression of oxidative stress-related kinases in monocytes from transgenic sheep. Detection of the expression levels of oxidative stress kinases: (a) COX-2, (b) iNOS, and (c) NADPH oxidase. Detection of the activation of antioxidative damage-related kinases: (d) T-SOD, (e) CAT, and (f) Gsh-Px. Data represent mean ± SE. ^a,b,c,d^Different superscripts indicate significantly different values between groups (*p* < 0.05). Wt, wild type; Tg-1, Tg-2, and Tg-3 were transgenic sheep that had 1, 2, or 3 copies of TLR4.

Oxidation intermediate products are major drivers of oxidative stress and are mostly either NO or O_2_^−^. Catalysis by COX-2 can promote NO synthesis and secretion. In this present study, monocytes with various exogenous TLR4 copy numbers were challenged with 1 *μ*g/mL LPS for 8 h. Compared with Wt sheep, activation of cellular COX-2 and iNOS was significantly higher in Tg animals (Figures 3(a) and 3(b)). Meanwhile, COX-2 and iNOS activation were well correlated with TLR4 copy number.

Enzyme activities between the transgenic groups were significantly different (*p* < 0.05). For NADPH oxidase activation, there was no significant difference between the Tg-1 and Wt groups; however, there was a significant difference between the Tg-2 and Tg-3 groups (*p* < 0.05). Levels of NADPH oxidase activation in the Tg-2 group were much higher than those in the Tg-1 and Wt groups (Figure 3(c)). These findings indicated that overexpressed TLR4 could trigger ROS/RNS release and could consequently induce oxidative stress. More copies of TLR4 enhanced free radical release and increased cellular oxidative stress.

### 3.3. In Response to LPS Stimulation, Overexpressed TLR4 Reduced T-SOD and Increased GSH-Px Activities

In this present study, monocytes that expressed various copy numbers of TLR4 showed similar patterns of T-SOD and CAT activation after 8 h exposure to LPS (Figures 3(d) and 3(e)). Expression levels in cells from Tg-1 sheep were higher than those from Wt sheep, but the differences were not significant (*p* > 0.05). Both of these enzymes exhibited lower expression levels in the Tg-3 group compared with the Tg-2 group (*p* < 0.05). GSH-Px activation tended to be positively correlated with TLR4 copy number (Figure 3(f)). We found that TLR4 promoted the activation of GSH-Px, T-SOD, and CAT at relatively low copy numbers to maintain the level of oxidative stress. Increased TLR4 copy numbers could reduce T-SOD and CAT activation, whereas GSH-Px activation was increased.

### 3.4. Reduced GSH Content in TLR4 Overexpressing Sheep

**Figure 4:**
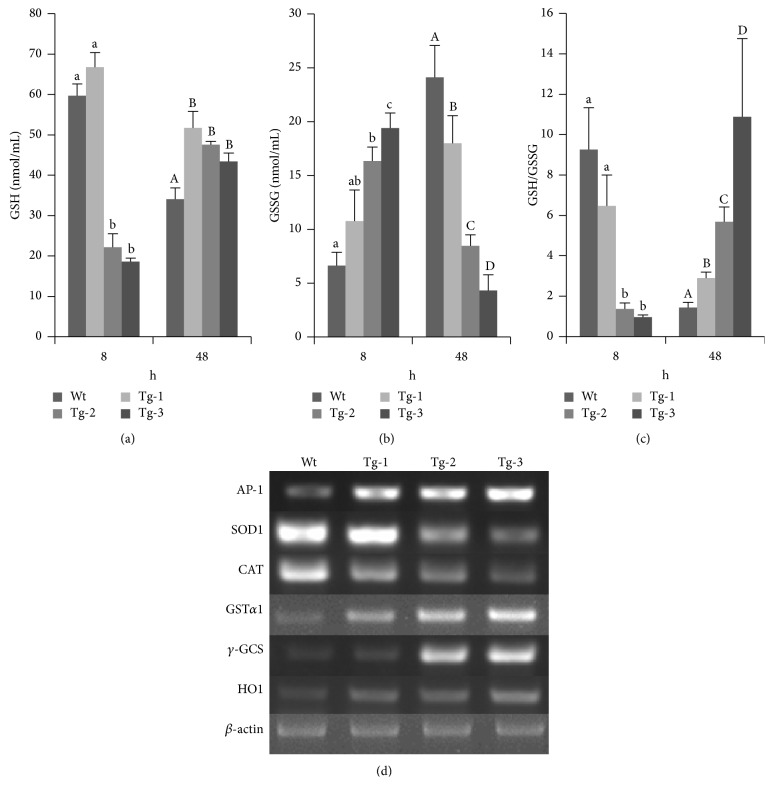
Contents of GSH and oxidative stress-related genes in TLR4 transgenic sheep monocytes/macrophages. (a) GSH content measurements, (b) GSSG content measurements, (c) GSH/GSSG ratio calculations, (d) RT-PCR detection of AP-1 and antioxidative-related gene expression. Data represent mean ± SE. ^a,b,c,d;A,B,C,D^Different superscripts indicate significantly different values between groups (*p* < 0.05). Wt, wild type; Tg-1, Tg-2, and Tg-3 were transgenic sheep that had 1, 2, or 3 copies of TLR4.

Monocytes with various TLR4 copy numbers were collected and challenged with LPS for 8 h. GSH and GSSG contents were measured (Figures 4(a) and 4(b)), and they were higher in the Tg-1 group, although there was no significant difference between the Tg-1 and Wt groups (*p* > 0.05). The GSSG contents tended to increase in correlation with the TLR4 copy number, which was in contrast to those of GSH. Overexpressed TLR4 caused more oxidative damage. During this process, oxidation intermediate products were scavenged. GSH was maintained at a higher level in transgene-positive monocytes, and this difference was significant when compared with the Wt group (*p* < 0.05). GSSG contents were negatively correlated with TLR4 copy number, indicating that the free radicals had been scavenged.

The ratio of GSH/GSSG indirectly reflects the levels of oxidative stress, so ratios were calculated for each group at both 8 and 48 h. We found that the ratio of GSH/GSSG was negatively correlated with TLR4 copy number and was lower in the Tg-2 and Tg-3 groups compared with the Tg-1 and Wt groups at 8 h (*p* < 0.05; Figure 4(c)). At 48 h after stimulation, the ratio of GSH/GSSG had returned to the average level. These findings indicated that, in response to LPS stimulation, extra copies of TLR4 could lead to lower amounts of GSH. However, over time, the ratio could quickly increase to an average level. The period of time over which oxidative damage occurred was reduced in transgenic animals.

### 3.5. Overexpression of TLR4 Promoted AP-1 Expression and the Regulation of Antioxidative Stress Genes

**Figure 5:**
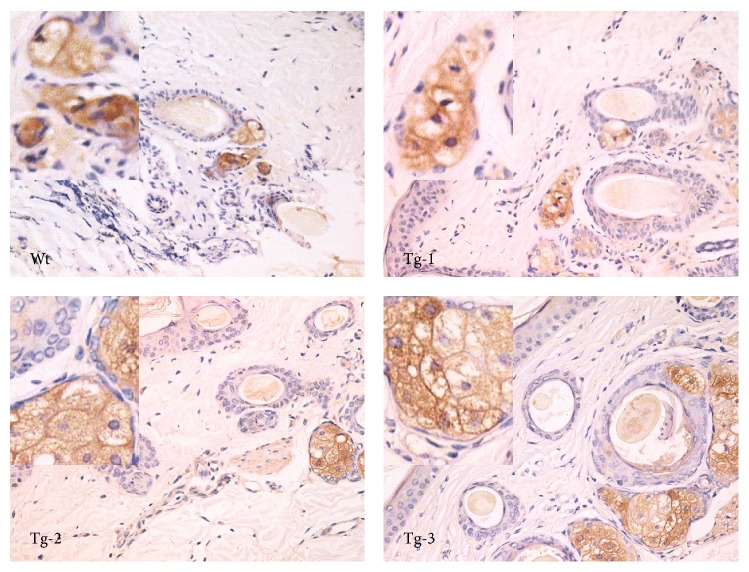
*γ*-GCS expression in TLR4 overexpressing sheep ear tissues. *γ*-GCS expression was detected by immunohistochemical staining (400x magnification is shown). Data represent mean ± SE. Wt, wild type; Tg-1, Tg-2, and Tg-3 were transgenic sheep that had 1, 2, or 3 copies of TLR4.

Real-time PCR was used to study TLR4 downstream genes expressed, including AP-1, CAT, SOD1, GST*α*1, *γ*-GCS, and HOl (Figure 4(d)). Levels of AP-1 expression increased in correlation with exogenous TLR4 copy numbers. The expression levels of GST*α*1, *γ*-GCS, and HOl showed similar patterns. By contrast, CAT and SOD1 expression levels showed a decreasing pattern. *γ*-GCS could also be observed by immunohistochemistry. More *γ*-GCS was observed in the high TLR4 copy number group (Figure 5). Levels of CAT and SOD1 expression were suppressed in the high TLR4 copy number groups. AP-1 expression levels were higher in the high TLR4 copy number groups. In support of a feedback regulation mechanism, HO1 expression was enhanced, which could potentially neutralize any oxidative effects. The increased amounts of GST*α*1 and *γ*-GCS expression indicated that GSH synthesis was promoted to scavenge free radicals.

## 4. Discussion

Both oxidative stress and LPS-induced immunity response can share TLR4 pathways. Binding with myeloid differentiation factor 2 (MD2) LPS induces TLR4 signal transduction, enhancing both phagocytosis and cytokine production in response to Gram-negative bacteria [17]. There are two major pathways for TLR4 transduction, MyD88-dependent and independent pathways. In the MyD88-dependent pathway, MyD88 triggers interleukin-1 receptor-associated kinase (IRAK) binding to TNF receptor-associated factor-6 (TRAF6), resulting in the nuclear translocation of NF-*κ*B, and initiates the AP-1 pathway [18, 19]. Meanwhile, TLR4 pathways play important roles in oxidative stress by promoting oxidative stress-related enzyme activation [20]. Products of oxidative stress act as second messengers to promote cytokine synthesis by activating NF-кB and AP-1 [21]. Since the iNOS is one of the downstream genes of TLR4, the TLR4 pathways contributed to upregulation of iNOS in transgenic sheep. Consequently, neutrophils were triggered to produce superoxide, which can increase macrophage activation [22]. COX-2 is the rate-limiting enzyme of prostaglandin synthesis, and COX-2 is involved in both acute and chronic inflammation under pathological conditions. Both NF-*κ*B and AP-1 can regulate COX-2 transcription [23]. In this study, COX-2 expression tended to increase according to TLR4 copies, which indicated that COX-2 transcription was regulated by TLR4 pathway. NAPDH oxidase production is administrated by TLR4 through the IRAK4 pathway [24]. We found that LPS activated AP-1 in transgenic animals, especially in sheep with high TLR4 copy numbers. This could be a result of AP-1 interacting with NF-кB [25]. Meanwhile, expression of HO1, another important anti-inflammatory enzyme, was detected. Results showed HO1 expressions were found increasing in transgenic sheep. Previous studies showed that HO1 directly regulates AP-1 expression, independently of its catalytic activity [26, 27]. Increased HO1 activity can suppress TLR4-induced signal transduction [28]. Our results suggest the negative feedback loop was initiated to reduce the inflammatory response in TLR4-overexpressed sheep.

Oxidative stress represents disequilibrium of the oxidative system. When an organ is infected, many types of inflammatory cells are activated. NO production is promoted and a large amount of oxygen free radicals is generated to remove pathogens. Large amounts of oxidation intermediates and their derivatives can not only destroy bacteria membranes, but also cause tissue damage [29]. A key feature of oxidative damage is the breakdown of the enzymatic defense system. SOD is a crucial enzyme for scavenging oxygen free radicals. In this present study, the SOD activation was found upregulated in Tg-1 animals. This suggests that the tissue was under mild oxidative stress conditions [30]. While SOD expression was suppressed in Tg-2 and Tg-3 groups, this indicated that tissues were under acute stress conditions. SOD is consumed in the process of scavenging oxygen free radicals. Subsequently, tissues are damaged by reactive oxygen accumulation [31]. All these findings indicated that excess SOD was consumed in TLR4 transgenic sheep and that overexpressed TLR4 caused serious damage to the antioxidative stress enzyme system in transgenic sheep.

Glutathione is a nonenzymatic antioxidant component that is important for organs to guard against free radicals. In the presence of GSH, GSH-Px can catalyze almost all ROOH into ROH. In certain tissues, GSH-Px acts in place of CAT to eliminate H_2_O_2_ [32]. In this present study, CAT activity was reduced and GSH-Px expression was increased following LPS stimulation. GST might regulate antioxidative enzyme expression [33] and AP-1 can upregulate GST transcription, whereas GSH can downregulate GST transcription [34]. We obtained similar results, as AP-1 expression was observed to increase, and levels of GST*α*1 transcription were also found to be elevated. In inflammatory conditions, ROS can be produced by macrophages and neutrophils. The inflammation caused by ROS could be reversed by the addition of exogenous GSH [35]. In this present study, expressions of *γ*-GCS, rate-limiting enzyme for GSH synthesis, were observed to be increasing correlated with TLR4 copy numbers. Under oxidative stress conditions, *γ*-GCS is compensatory upregulated to increase GSH synthesis. GSH can be transferred to GSSG to eliminate free radicals avoiding tissue's oxidative damage [36, 37]. But the extra copies of the TLR4 gene led to more rapid GSH consumption and dramatically increased GSSG. This finding suggests that more severe oxidative damage occurred in TLR4 overexpressing sheep. Over time, GSH was maintained at a relatively high level and returned to an average level more quickly in transgenic animals. Accordingly, the time period of oxidative damage was shortened.

## 5. Conclusions

It has been established that TLR4 is related to the oxidative stress response [38]. This present study found that overexpressed TLR4 inhibited SOD activity and triggered AP-1 to initiate downstream antioxidative genes that protect against oxidative stress. We found that overexpressed TLR4 increased antioxidative stress capacity. TLR4 promoted AP-1 expression, and subsequently *γ*-GCS expression was upregulated to maintain tissue homeostasis.

## Figures and Tables

**Table 1:** The primer sequences.

Primer	Forward (5′-3′)	Reverse (5′-3′)
cTLR4	TACGGTAAACTGCCCACTTG	ACCTGGAGAAGTTATGGCTG
TLR4	CTGAATCTCTACAAAATCCC	CTTAATTTCGCATCTGGATA
AP-1	TGAAGGAAGAGCCGCAGAC	CCACCTGTTCCCTGAGCATA
SOD1	CGAGGCAAAGGGAGATAAA	CTCCAGCGTTTCCAGTCTT
CAT	GAAACGCCTGTGTGAGAAC	ACATAGGTGTGAACTGCGT
GST*α*1	GTTCCAGCAAGTGCCAATG	GGGAGATAACGGTTTGTCG
*γ*-GCS	ATGGCTCAAGCGTTCGTCA	CAGTTCCCCTCTCTCGTGC
HO1	GGCGGAGAATGCCGAGTT	CCTCCTGGAGTCGCTGAACAT
*β*-actin	AGATGTGGATCAGCAAGCAG	CCAATCTCATCTCGTTTTCTG
